# Effectiveness of controlled-expansion transjugular intrahepatic portosystemic shunt (CX-TIPS) in an interdisciplinary setting at a large tertiary center

**DOI:** 10.1007/s00508-025-02606-7

**Published:** 2025-09-04

**Authors:** Marlene Hintersteininger, Julia Kappel, Theresa Müllner-Buscics, Susanna Riegler, Nina Dominik, Georg Kramer, Christian Sebesta, Paul Thöne, Albert Friedrich Stättermayer, Lukas Reider, Maria Schoder, Catharina Klausenitz, Raoul Varga, Fredrik Waneck, Michael Trauner, Mattias Mandorfer, Thomas Reiberger, Lukas Hartl

**Affiliations:** 1 Division of Gastroenterology and Hepatology, Department of Medicine III, Medical University of Vienna, Vienna, Austria; 2 Vienna Hepatic Hemodynamic Lab, Division of Gastroenterology and Hepatology, Department of Medicine III, Medical University of Vienna, Vienna, Austria; 3 Clinical Research Group MOTION, Medical University of Vienna, Vienna, Austria; 4 Christian Doppler Lab for Portal Hypertension and Liver Fibrosis, Medical University of Vienna, Vienna, Austria; 5 Division of Cardiovascular and Interventional Radiology, Department of Biomedical Imaging and Image-guided Therapy, Medical University of Vienna, Vienna, Austria

**Keywords:** Collaterals, Portal hypertension, Survival, Interdisciplinary board, Ascites, Variceal bleeding

## Abstract

**Introduction:**

The use of controlled-expansion transjugular intrahepatic portosystemic shunt (CX-TIPS) effectively controls portal hypertension (PH)-related complications while reducing risks related to fully expanded stents. We evaluated the effectiveness of CX-TIPS in a large Viennese patient cohort.

**Method:**

We assessed the number of patients evaluated for CX-TIPS placement by interdisciplinary discussion at the Medical University of Vienna and included all patients from the prospective AUTIPS registry undergoing CX-TIPS placement between June 2018 – December 2024. After clinical and laboratory characterization at baseline, patients were followed up for clinical events.

**Results:**

Overall, 200 patients underwent interdisciplinary evaluation for CX-TIPS. In 62.5% CX-TIPS was recommended and 82.4% of these patients underwent CX-TIPS placement. Among 103 patients with CX-TIPS placement between June 2018 – December 2024 (median age 57 years, 67.0% male, median MELD 11), the primary indication for CX-TIPS implantation was ascites (65.0%). The median portal pressure gradient decreased from 18 mm Hg to 8 mm Hg after CX-TIPS. Underdilated CX-TIPS placement was performed in 13.6% (*n* = 14) of patients and portal vein recanalization (PVR-TIPS) was performed in 4 patients. During a median follow-up time of 13.5 months, 30.1% of patients experienced hepatic encephalopathy and 5.8% cardiac decompensation. Kaplan-Meier survival analyses revealed 1‑year and 3‑year transplant-free survival rates of 78.0% and 74.7%, respectively.

**Conclusion:**

Implementation of interdisciplinary case discussions and the use of CX-TIPS enable personalized medicine in patients with cirrhosis. Underdilation of CX-TIPS should be considered in patients at high risk for complications.

**Supplementary Information:**

The online version of this article (10.1007/s00508-025-02606-7) contains supplementary material, which is available to authorized users.

## Introduction

Liver cirrhosis is associated with considerable morbidity and mortality worldwide [1]. The decompensated stage is characterized by the development of complications of portal hypertension (PH), such as variceal bleeding and ascites [2–4]. Placement of transjugular intrahepatic portosystemic shunt (TIPS) is an effective treatment option for patients with these complications by lowering portal pressure [3, 5], decreasing systemic inflammation [6, 7] and improving clinical outcomes [8]. Specifically, TIPS lowers mortality in patients with refractory and recurrent ascites [9, 10] and in patients with severe variceal bleeding [11, 12].

In the early stages, bare metal stents (BMS) were used to connect a branch of the portal vein with a liver vein [13]. These stents were available in Vienna starting in 1991 [14]; however, BMS were associated with suboptimal clinical outcomes including stent stenosis or occlusion in more than 50% of patients within 1 year [15]. The subsequent development of expandable polytetrafluoroethylene (ePTFE)-covered stents significantly improved medical outcomes, as it was associated with higher shunt patency rates [16] and improved survival compared to BMS TIPS [17].

The recent introduction of covered controlled expansion TIPS (CX-TIPS) represents further progress, as they allow stent dilation up to its nominal diameter (usually between 8 and 10 mm) without further passive dilation [18]. This is significant, as the current guidelines recommend to have the smallest necessary TIPS diameter to achieve adequate hemodynamic response [19] and to minimize complications of TIPS such as hepatic encephalopathy or cardiac decompensation. Underdilation of TIPS is possible and has already been investigated in a number of studies, suggesting a passive dilation of the stent to its nominal diameter over time [19–22].

Given the complexity of PH and the potential risks associated with TIPS placement, an interdisciplinary approach may be beneficial. Inspired by oncological models [23], an interdisciplinary PH/TIPS board has been established at our institution to discuss the technical feasibility and clinical appropriateness of CX-TIPS on a case-by-case basis.

This study aimed to illuminate the current clinical practice concerning CX-TIPS in a large Viennese center by (i) reporting the number of patients evaluated for CX-TIPS placement by interdisciplinary discussion and assessing how many underwent CX-TIPS placement. Moreover, we (ii) investigated patient characteristics and clinical outcomes in patients undergoing CX-TIPS placement with a particular focus on underdilated CX-TIPS.

## Patients and methods

### Study design

This is an exploratory, single-center cohort study with retrospective assessment of prospectively collected data. Since 2018, placement of CX-TIPS has been routinely conducted at the Vienna General Hospital. This study included patients with chronic liver disease undergoing elective CX-TIPS placement for the indication of refractory ascites or portal hypertensive bleeding between June 2018 and December 2024, who were included in the prospective AUTIPS registry (NCT: NCT03409263). The stent diameters ranged from 6–10 mm, allowing for classification into underdilated CX-TIPS (defined as dilation to a diameter of 6–7 mm) and non-underdilated CX-TIPS (defined as a diameter of 8–10 mm).

Furthermore, the study cohort was compared to a historical control group of patients who underwent elective ePTFE-TIPS placement between January 2010 and May 2018.

### Interdisciplinary case discussions

At the Vienna General Hospital, an interdisciplinary PH/TIPS board has been instituted. Patients considered for TIPS placement could be referred to the board/meeting by all physicians. The board convened monthly, bringing together hepatologists and interventional radiologists to evaluate each case individually and to determine the technical and clinical feasibility of TIPS placement.

### CX-TIPS procedure

The CX-TIPS was carried out in accordance with the standard operating procedures (SOPs) of the Vienna General Hospital. The intervention was performed under sterile conditions by an experienced interventional radiologist, utilizing the Seldinger technique to introduce a catheter into a hepatic vein. Portal pressure gradient (PPG) was measured before CX-TIPS placement. Following successful puncture of the portal vein, parenchymal tract dilation was performed using a balloon catheter. Subsequently, a VIATORR® TIPS Endoprosthesis with controlled expansion (W.L. Gore & Associates, Inc., Newark, DE, USA) was implanted in the CX-TIPS group and individually dilated, while the ePTFE-TIPS group received a PTFE-covered TIPS with a given nominal diameter. Finally, the PPG was reassessed.

### Laboratory assessment

All laboratory tests were conducted at the ISO-certified Department of Laboratory Medicine of the Vienna General Hospital. Blood samples were obtained as part of routine clinical care and were analyzed at three predefined time points: (i) 1 day prior to CX-TIPS implantation, (ii) 3 months post-intervention, and (iii) 1 year following CX-TIPS placement.

### Assessment of follow-up

Clinical events occurring during follow-up (FU) were systematically recorded, including the development of decompensation events after CX-TIPS placement (such as worsening of ascites, variceal bleeding, and overt hepatic encephalopathy) as well as liver transplantation and liver-related mortality. Acute on chronic liver failure (ACLF) was defined following the EASL/EF-CLIF definition [24]. In addition, the occurrence of cardiac decompensation (defined as clinical signs of hypervolemia, such as moderate or severe leg edema or pleura effusions due to cardiac insufficiency), TIPS thrombosis and the need for reintervention to either expand or reduce the stent diameter were systematically documented.

### Statistical analysis

Categorical variables were summarized as absolute numbers (*n*) and relative frequencies (%). Continuous variables were presented as medians with interquartile ranges (IQR). Comparisons of continuous, non-normally distributed variables between two groups were performed using the Mann-Whitney U test, while the Kruskal-Wallis test was applied for comparisons across three or more groups. For unpaired categorical variables, Pearson’s χ^2^-test was employed. Data were visualized using histograms.

To assess overall survival Kaplan-Meier survival analysis was performed and survival curves were generated. Differences between groups were evaluated using the log-rank test.

Statistical analyses were performed using IBM SPSS Statistics 27.0 (IBM, Armonk, NY, USA), R 4.2.1 (R Core Team, R Foundation for Statistical Computing, Vienna, Austria), and GraphPad Prism 8 (GraphPad Software, La Jolla, CA, USA). A two-sided *p*-value of < 0.05 was considered statistically significant.

### Ethics

The study was approved by the ethics committee (EC) of the Medical University of Vienna (EK 1760/2014; EK 1943/2017) and performed according to the current vision of the Helsinki Declaration. All patients who underwent CX-TIPS placement were part of the prospective register study at the Medical University of Vienna (AUTIPS; NCT: NCT03409263). The EC waived the need for written informed consent for the retrospectively included patients.

## Results

### Interdisciplinary case discussions

**Fig. 1:**
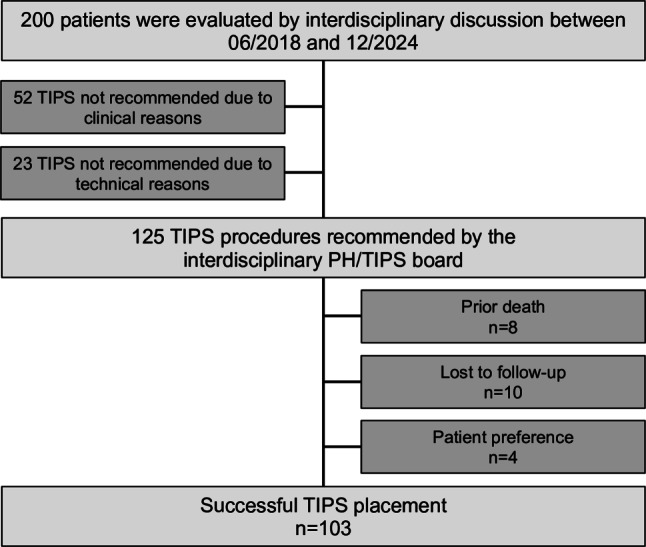
Patient flow chart. *n* number; *PH* portal hypertension; *TIPS* transjugular intrahepatic portosystemic shunt

Between June 2018 and December 2024 a total of 200 patients were evaluated for CX-TIPS placement by interdisciplinary discussion (Fig. 1). Among these patients, CX-TIPS was regarded as technically unfeasible in 11.5% (*n* = 23/200) following the evaluation of CT imaging and was not recommended due to clinical reasons in 26.0% (*n* = 52/200). In patients for whom CX-TIPS was recommended, 82.4% (*n* = 103/125) underwent CX-TIPS procedure, while no CX-TIPS was placed in 4 patients (3.2%) due to patient preference, in 10 patients (8.0%) as they were lost to follow-up before CX-TIPS placement and 8 patients (6.4%) died prior to CX-TIPS placement.

### Patient characteristics

A total of 103 patients enrolled in the prospective AUTIPS registry underwent successful CX-TIPS placement between June 2018 and December 2024. Baseline characteristics of these patients compared to the historic control group (*n* = 98) are shown in Table 1. Most of the patients were male (67%) and median age was 57 years. Alcohol-related liver disease (ALD) was the most common underlying etiology (61.2%). The primary indication for CX-TIPS implantation was ascites (65.0%), whereas portal hypertension-related bleeding was the indication in 35.0% of patients. In most cases (70.9%), the CX-TIPS was dilated to a diameter of 8 mm, while a larger diameter (i.e., 9–10 mm) was used in 15.5% (*n* = 16) and a smaller one (i.e., underdilated to 6–7 mm) in 13.6% (*n* = 14). The median portal pressure gradient decreased from baseline 18 mm Hg to 8 mm Hg after CX-TIPS placement. If the PPG was decreased to < 10 mm Hg, non-selective beta blockers for portal hypertension indication were discontinued and 86 patients received HE prophylaxis after TIPS placement (specifically *n* = 79 rifaximin, *n* = 64 lactulose and *n* = 36 L‑ornithine-L-aspartate).

**Table 1:** Patient characteristics at the time of CX-TIPS placement

Patient characteristics	CX-TIPS cohort (*n* = 103)	ePTFE-TIPS cohort (*n* = 98)	p-value
*Sex, male/female (% male)*	69/34 (67.0%)	68/30 (69.4%)	0.715
*Age, years (IQR)*	57.0 (49.0–66.0)	56.0 (49.8–63.0)	0.695
*BMI, kg* *×* *m*^*2*^* (IQR)*	25.5 (22.0–29.4)	25.3 (22.1–28.7)	0.941
*Etiology*			0.774
– *ALD, n (%)*	63 (61.2%)	58 (59.2%)	
– *Non-ALD, n (%)*	40 (38.8%)	40 (40.8%)	
*MELD, points (IQR)*	11.2 (9.5–15.7)	13.0 (9.8–18.5)	0.086
*CTP score, points (IQR)*	8.0 (7.0–9.0)	8.0 (7.0–9.0)	0.833
*CTP stage*			0.273
– *A, n* (%)	16 (15.5%)	21 (21.4%)	
– *B, n* (%)	75 (72.8%)	66 (67.4%)	
– *C, n* (%)	12 (11.7%)	11 (11.2%)	
*Ammonia, µmol* *×* *L*^*-1*^* (IQR)*	41.1 (29.1–58.8)	48.9 (36.1–67.0)	**0.024**
proBNP*, pg *×* *mL*^*-1*^* (IQR)*	203.0 (96.4–536.8)	–	–
*TIPS Indication*			0.988
– *Bleeding, n (%)*	36 (35.0%)	34 (34.7%)	
– *Ascites, n (%)*	67 (65.0%)	63 (64.3%)	
*TIPS diameter, (IQR)*	8.0 (8.0–8.0)	8.0 (8.0–9.0)	**0.001**
– *6 mm, n (%)*	3 (2.9%)	0 (0.0%)	
– *7 mm, n (%)*	11 (10.7%)	0 (0.0%)	
– *8 mm, n (%)*	73 (70.9%)	69 (70.4%)	
– *9 mm, n (%)*	6 (5.8%)	8 (8.2%)	
– *10 mm, n (%)*	10 (9.7%)	21 (21.4%)	
*PPG pre-TIPS, mm* *Hg (IQR)*	18.0 (15.0–22.0)	20.0 (16.0–25.0)	0.089
*PPG post-TIPS, mm* *Hg (IQR)*	8.0 (6.0–10.0)	7.0 (6.0–9.0)	0.178

*ALD* alcohol-related liver disease; *CTP* Child-Turcotte-Pugh score; *CX* controlled-expansion; *BMI* body mass index; *ePTFE* expandable polytetrafluoroethylene; *IQR* interquartile range, *MELD* model of end-stage liver disease; *PPG* portal pressure gradient; *TIPS* transjugular intrahepatic portosystemic shunt

*proBNP levels were not available in the ePTFE-TIPS cohort

### Underdilated CX-TIPS placement

Table S1 details the patient characteristics of patients with underdilated CX-TIPS as compared to patients with non-underdilated CX-TIPS. Overall, 14 patients (13.6%) received an underdilated CX-TIPS with a stent diameter of 6 (*n* = 3) or 7 mm (*n* = 11). The reasons for underdilation were previous episodes of hepatic encephalopathy (*n* = 3), cardiac risk factors (*n* = 6) or sarcopenia (*n* = 5). In half of these patients (*n* = 7/14) the etiology of liver disease was ALD. The main indication was ascites (*n* = 12/14), while in two cases CX-TIPS placement was performed due to bleeding. No significant differences in the baseline characteristics were observed between patients with underdilated CX-TIPS and those with a CX-TIPS diameter between 8 and 10 mm; however, patients with underdilated CX-TIPS tended to have a higher Child-Pugh stage (stage C: underdilated 21.4% vs. non-underdilated 10.1%), although this difference was not statistically significant (*p* = 0.296). Prior to CX-TIPS implantation, four patients had overt hepatic encephalopathy (West-Haven grade I or II). The remaining 10 cases did not exhibit hepatic encephalopathy before CX-TIPS placement. The PPG before and after CX-TIPS placement was comparable in patients with underdilated and non-underdilated CX-TIPS. Notably, there were no procedure-related complications in patients with underdilated CX-TIPS.

### PVR-TIPS placement

In 4 out of 103 patients (3.8%), a portal vein recanalization-transjugular intrahepatic portosystemic shunt (PVR-TIPS) was performed due to chronic portal vein thrombosis that prevented access via the transjugular route. Characteristics of patients with PVR-TIPS placement are shown in Table 2. Of the patients two suffered from cryptogenic liver cirrhosis, one had a mixed etiology of ALD and viral hepatitis, and the fourth patient had non-cirrhotic clinically significant portal hypertension secondary to portal vein thrombosis. In all four cases, the indication for PVR-TIPS placement was gastrointestinal bleeding. Stent dilation was performed to 8 mm in 2 patients and to 9 mm in the remaining 2 cases.

**Table 2:** Baseline characteristics of patients with PVR-TIPS placement

Patient characteristics	PVR-TIPS (*n* = 4)
*Sex, male/female (% male)*	3/1 (75.0%)
*Age, years (IQR)*	64.5 (41.5–71.0)
*BMI, kg* *×* *m*^*2*^* (IQR)*	28.6 (25.2–33.2)
*Etiology*	
– *ALD + viral hepatitis, n (%)*	1 (25.0%)
– *Cryptogenic, n (%)*	2 (50.0%)
– *Other, n (%)*	1 (25.0%)
*MELD, points (IQR)*	9.4 (6.7–11.6)
*CTP score, points (IQR)*	6.0 (5.3–7.5)
*CTP stage*	
– *A, n (%)*	3 (75.0%)
– *B, n (%)*	1 (25.0%)
– *C, n (%)*	0 (0.0%)
*TIPS Indication*	
– *Bleeding, n (%)*	4 (100.0%)
– *Ascites, n (%)*	0 (0.0%)
*TIPS diameter*	
– *8 mm, n (%)*	2 (50.0%)
– *9 mm, n (%)*	2 (50.0%)

*ALD* alcohol-related liver disease; *CTP* Child-Turcotte-Pugh-Score; *BMI* body mass index; *IQR* interquartile range *MELD* model of end-stage liver disease, *PVR* portal vein recanalization; *TIPS* transjugular intrahepatic portosystemic shunt

### Laboratory data

**Fig. 2:**
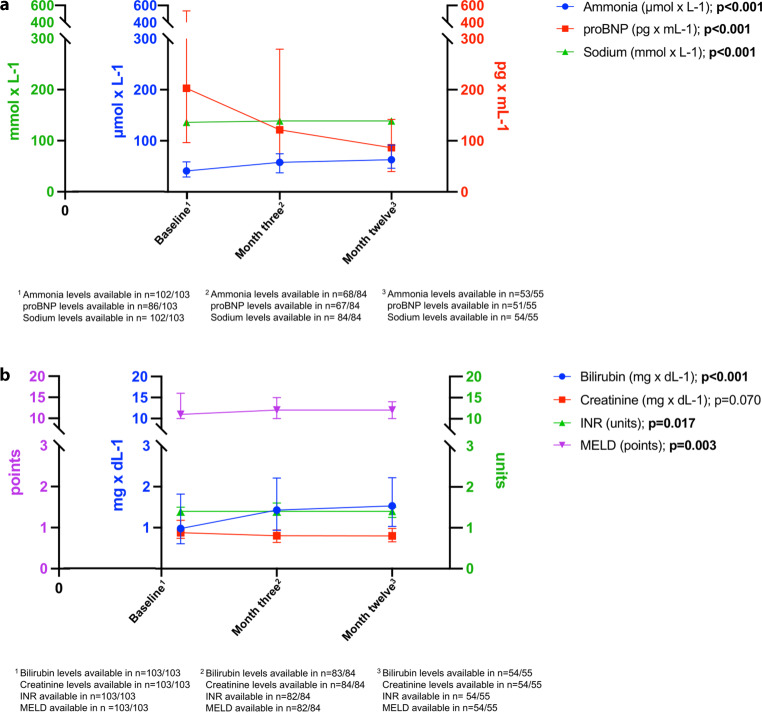
Course of biomarkers and MELD components after CX-TIPS placement. (**a**) Showing ammonia, proBNP and sodium and (**b**) showing bilirubin, creatinine, INR and MELD. *MELD* model for end-stage liver disease, *proBNP* brain natriuretic peptide

Changes in laboratory values from baseline to 1 year following CX-TIPS placement are shown in Table 3 and Fig. 2. The median levels of ammonia significantly increased from 41.1 µmol/L (IQR 29.1–58.8) to 57.7 µmol/L (IQR 37.3–74.5) at 3 months and 62.9 µmol/L (IQR 46.2–91.6) at 12 months after CX-TIPS placement (*p* < 0.001). No correlation could be found between the change in ammonia levels from BL to month 3 and PPG reduction (ρ = 0.052, *p* = 0.698). Furthermore, there was a slight increase of median bilirubin (1.0 mg/dL [IQR 0.6–1.8] at baseline versus 1.4 mg/dL [IQR 1.0–2.2] after 3 and 1.5 mg/dL [IQR 1.0–2.2] after 12 months; *p* < 0.001), sodium (136.0 mmol/L [IQR 133.0–139.0] at baseline versus 139.0 mmol/L [IQR 136.0–141.0] after 3 and 139.0 mmol/L [IQR 137.5–141.0] after 12 months; *p* < 0.001) and albumin (34.6 g/L [IQR 31.3–37.9] at baseline versus 35.6 g/L [IQR 31.6–39.3] after 3 and 38.9 g/L [IQR 35.9–41.2] after 12 months; *p* < 0.001) after CX-TIPS implantation. Interestingly, median proBNP levels significantly decreased from 203.0 pg/mL (IQR 96.4–536.8) to 121.5 pg/mL (IQR 56.3–279.5) after 3 months and to 81.3 pg/mL (IQR 39.8–152.8) after 12 months (*p* < 0.001).

**Table 3:** Laboratory course after CX-TIPS implantation

Parameter	At baseline	3 months after CX-TIPS implantation	1 year after CX-TIPS implantation	p‑value*
Patients	*n* = 103	*n* = 84	*n* = 55	*–*
Bilirubin, mg × dL^-1^ (IQR)	1.0 (0.6–1.8)	1.4 (1.0–2.2)	1.5 (1.0–2.2)	***< 0.001***
Albumin, g × dL^-1^ (IQR)	34.6 (31.3–37.9)	35.6 (31.6–39.3)	38.9 (35.9–41.2)	***< 0.001***
INR, units (IQR)	1.4 (1.3–1.5)	1.4 (1.3–1.6)	1.4 (1.2–1.5)	***0.017***
Creatinine, mg × dL^-1^ (IQR)	0.9 (0.7–1.2)	0.8 (0.6–0.9)	0.8 (0.7–1.0)	*0.070*
Sodium, mmol × L^-1^ (IQR)	136.0 (133.0–139.0)	139.0 (136.0–141.0)	139.0 (137.5–141.0)	***< 0.001***
Platelets, 10^9^ × L^-1^ (IQR)	121.0 (79.0–177.0)	112.0 (74.3–163.5)	104.0 (76.0–163.0)	*0.149*
WBC, G × L^-1^ (IQR)	5.7 (3.7–7.1)	5.4 (4.0–6.5)	5.4 (4.0–6.6)	***0.031***
CRP, mg × dL^-1^ (IQR)	0.8 (0.4–1.7)	0.5 (0.2–1.6)	0.4 (0.2–1.1)	***0.020***
Ammonia, µmol × L^-1^ (IQR)	41.1 (29.1–58.8)	57.7 (37.3–74.5)	62.9 (46.2–91.6)	***< 0.001***
proBNP, pg × mL^-1^ (IQR)	203.0 (96.4–536.8)	121.5 (56.3–279.5)	81.3 (39.8–152.8)	***< 0.001***
MELD, points (IQR)	11.2 (9.5–15.7)	12.4 (10.3–14.8)	12.3 (10.3–14.5)	***0.003***

*CRP* C-reactive protein; *IQR* interquartile range; *INR* international normalized ratio, *MELD* model of end-stage liver disease,; *proBNP* brain natriuretic peptide; *WBC* white blood cell count

*The *p*-values indicate the statistical significance of the differences between all three groups

### Follow-up and clinical outcomes

The median FU time was 13.5 months. Table 4 provides an overview of clinical outcome parameters in this cohort compared to the control group. Overall, 43.7% (*n* = 45/103) of the patients experienced at least one hepatic decompensation event, of which hepatic encephalopathy (30.1%) and ascites complication (29.1%) were the most frequent. Notably, patients who received an underdilated CX-TIPS experienced hepatic decompensation events significantly more frequently than those without an underdilated CX-TIPS (71.4% vs. 39.3%; *p* = 0.024). Regarding the individual complications of hepatic decompensation, variceal bleeding was the only event that differed significantly between the two groups, occurring in 14.3% of patients with underdilated CX-TIPS compared to 4.5% of those without underdilated CX-TIPS (*p* = 0.006). Hepatic encephalopathy was observed in 28.1% of patients without underdilated CX-TIPS and in 42.9% of those with underdilated CX-TIPS (*p* = 0.263; Figure S1A). No difference in ascites or bleeding control was observed during follow-up between the two groups (*p* = 0.336; Figure S1B). Of the patients 26.2% (*n* = 27/103) developed ACLF and 11 patients (10.7%) underwent liver transplantation. Only 5.8% (*n* = 6/103) experienced cardiac decompensation with no significant difference between patients with underdilated versus non-underdilated CX-TIPS (14.3% versus 4.5%; *p* = 0.146). A total of 18 patients (17.5%) required reintervention. In 13 cases (12.6%), further CX-TIPS dilation was performed, while 5 (4.9%) required diameter reduction due to hepatic encephalopathy. A CX-TIPS thrombosis occurred in seven patients, all of whom had initially received a non-underdilated CX-TIPS. Notably, nearly half of these cases (*n* = 3/7) involved patients who had undergone PVR-TIPS placement: however, during a median follow-up time of 36.0 months, none of the PVR-TIPS patients had additional episodes of portal hypertensive bleeding or developed hepatic encephalopathy and all were still alive at the end of follow-up.

**Table 4:** Follow-up and clinical outcomes

Clinical outcomes	CX-TIPS cohort (*n* = 103)	ePTFE-TIPS cohort (*n* = 98)	p-value
*Follow-up time, days (IQR)*	405 (176–827)	921 (263–2419)	**<** **0.001**
*Stent dilatation, n (%)*	13 (12.6%)	5 (5.1%)	0.062
*Stent reduction, n (%)*	5 (4.9%)	9 (9.2%)	0.228
CX-TIPS thrombosis*, n (%)	7 (6.8%)	–	–
*Decompensation event, n (%)*	45 (43.7%)	60 (61.2%)	**0.019**
– *Ascites complication, n* (%)	30 (29.1%)	37 (37.7%)	0.287
– *Variceal bleeding, n* (%)	3 (2.9%)	7 (7.1%)	0.168
– *Hepatic encephalopathy, n* (%)	31 (30.1%)	44 (44.9%)	**0.030**
*ACLF, n (%)*	27 (26.2%)	29 (29.6%)	0.593
*Cardiac decompensation, n (%)*	6 (5.8%)	14 (14.3%)	**0.045**
*SBP, n (%)*	4 (3.9%)	1 (1.0%)	0.193
*AKI, n (%)*	13 (12.6%)	35 (35.7%)	**<** **0.001**
*HCC, n (%)*	1 (1.0%)	5 (5.1%)	0.085
*Liver transplantation, n (%)*	11 (10.7%)	18 (18.4%)	0.121
*Death, n (%)*	25 (24.3%)	45 (45.9%)	**0.001**
*Liver-related death, n (%)*	18 (17.5%)	33 (33.7%)	**0.008**
*3‑month survival*	88.8%	91.6%	0.542
*1‑year survival*	78.0%	78.5%	0.860
*3‑year survival*	74.7%	61.1%	0.075

*ACLF* acute on chronic liver failure; *AKI* acute kidney injury; *CX* controlled expansion; *ePTFE* expandable polytetrafluoroethylene; *HCC* hepatocellular carcinoma; *IQR* interquartile range; *n* number; *SBP* spontaneous bacterial peritonitis; *TIPS* transjugular intrahepatic portosystemic shunt

*Data on CX-TIPS thrombosis were not available in the ePTFE-TIPS cohort

### Survival of patients with elective CX-TIPS placement

**Fig. 3:**
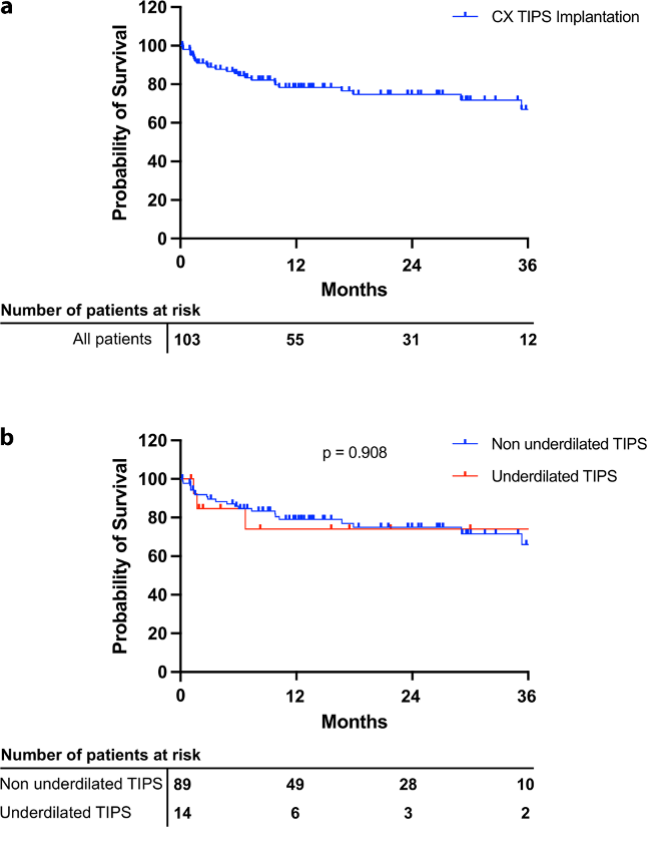
Survival after CX-TIPS implantation showing (**a**) all patients undergoing CX-TIPS implantation and (**b**) differentiating between patients undergoing underdilated versus non-underdilated TIPS placement. *CX* controlled expansion; *TIPS* transjugular intrahepatic portosystemic shunt

Out of 103 patients 25 (24.3%) died during follow-up, of which 72.0% were liver related deaths. Of the 25 cases 3 (12.0%) had received an underdilated CX-TIPS. Median transplant-free survival was 6 years. As shown in Fig. 3a and Table 4, 3‑month, 1‑year and 3‑year transplant-free survival rates were 88.8%, 78.0% and 74.7%, respectively. Patients with underdilated CX-TIPS did not differ in survival rates to patients with non-underdilated CX-TIPS placement (Fig. 3b, *p* = 0.908).

## Discussion

This study portrays current clinical practice concerning CX-TIPS placement in patients with chronic liver disease in a large Viennese center. We demonstrate that the interdisciplinary case discussions and establishment of a PH/TIPS board represent an opportunity for hepatologists and interventional radiologists to regularly assess potential TIPS candidates concerning technical feasibility and clinical appropriateness of TIPS. In patients undergoing CX-TIPS placement the median portal pressure gradient decreased from 18 to 8 mm Hg, representing hemodynamic success according to current guidelines [19]. Underdilation was performed in 13.6% and was not associated with any technical complications. Notable clinical complications after CX-TIPS included hepatic encephalopathy and ascites. Cardiac decompensation was a relatively rare event. Moreover, interdisciplinary discussion is the key to finding solutions in complex cases, as exemplified by the four patients with portal vein thrombosis and prior portal hypertensive bleeding who underwent successful PVR-TIPS placement. While 75% of these patients experienced at least partial thrombosis of the shunt during follow-up, there were no bleedings, episodes of hepatic encephalopathy or deaths in any of these patients.

In oncology, interdisciplinary tumor boards are well-established as valuable tools to adapt the current guidelines to the individual patient and represent the standard of care [23]. For this, experts of different specialties meet regularly to discuss oncology patients typically when they are diagnosed initially, as well as when they experience recurrence or progression of disease and before and after surgery [25]. Analogously, a PH/TIPS board has been established at the Medical University of Vienna. In this interdisciplinary board, which comes together monthly, individual patients with complications of PH are discussed, mainly regarding the technical feasibility and clinical appropriateness of TIPS placement, and the interventions are planned. Potential advantages include that this interdisciplinary board builds on the complementary expertise that is required for the increasingly complex patient characteristics of patients with decompensated cirrhosis (e.g., aged patients, higher prevalence of cardiometabolic risk factors). Moreover, an individualized strategy for implantation and the desired initial TIPS diameter can be discussed in direct communication with the case managers (potentially involving also other disciplines than hepatology) and interventional radiology.

Our data show that the PH/TIPS board has been utilized amply, as meanwhile 200 individual patients have been discussed. Notably, these discussions resulted in 103 patients undergoing CX-TIPS placement, representing four fifths of the patients, for whom CX-TIPS was recommended. Notably, the most frequent reasons for patients not undergoing CX-TIPS after recommendation were lost to follow-up, patient preference and death prior to CX-TIPS placement. We are confident that the interdisciplinary PH/TIPS board represents a milestone towards individualized medicine in patients with decompensated cirrhosis.

Moreover, this study investigated the characteristics and clinical as well as laboratory trajectories of patients undergoing CX-TIPS placement. Notably, this timeframe included the coronavirus disease 2019 (COVID-19) pandemic, which particularly limited patient access during the early phase [26]. Nevertheless, over 100 patients were enrolled. With a median MELD of 11 and a median Child-Pugh score of 8 at baseline, our patient cohort suffered from moderately impaired liver function, consistent with these being mostly stable patients, who underwent elective CX-TIPS placement. Notably, non-selective beta blockers were usually discontinued if the PPG was decreased to < 10 mm Hg. A recent study did not show any benefits of non-selective beta blockers after TIPS placement [27].

While after CX-TIPS placement, serum ammonia and bilirubin increased, interestingly, there was a significant decrease in proBNP after CX-TIPS, suggesting improved hemodynamic conditions, likely reflecting a decreased volume overload. Indeed, cardiac decompensation was an uncommon event during follow-up, experienced by only 5.8% of patients. We want to emphasize the importance of adequate patient selection [28], the basis of which is provided by a standardized TIPS evaluation, including transthoracic echocardiography and even right heart catheterization for elective CX-TIPS placement at our center. While the Toulouse algorithm has been proposed for cardiac risk stratification of patients after TIPS [28], it could not be validated by a recent study from Hannover, which found that diastolic dysfunction was linked to cardiac decompensation in this patient cohort [29].

Overall, the clinical outcomes were good with a 3-year transplant-free survival rate of 74.7%, as compared to 57.0% reported by a Viennese study in 2004 [14] and to 61.1% in the historic ePTFE-TIPS control cohort.

Approximately 30% of patients experienced overt hepatic encephalopathy during follow-up, which was comparable to international studies [30, 31]. Interestingly, the rate of hepatic encephalopathy was numerically higher among patients with underdilated CX-TIPS, possibly reflecting that such patients are typically deemed high-risk patients even before CX-TIPS placement. In line, the number of hepatic decompensation events was higher among patients with underdilated CX-TIPS, although we want to emphasize that mortality was not different between the groups. This could even mean that high-risk patients profit relatively more from underdilated CX-TIPS interventions than stable patients undergoing normal-diameter CX-TIPS. In this context, we also want to highlight an Italian study, which reported clinical feasibility and actually lower rates of portosystemic hepatic encephalopathy among patients with underdilated CX-TIPS [32].

Finally, our study included four patients with successful placement of PVR-TIPS. This indicates the technical feasibility of this procedure at a large specialized center; however, the high rate of thrombosis after PVR-TIPS placement indicates the need for anticoagulation in these patients.

Our study also has limitations. This is a unicentric study and thus requires external validation, although we want to emphasize that our data are well in line with current international studies. Moreover, our sample size is limited and further research is required, particularly to determine the value of underdilated CX-TIPS and PVR-TIPS. Finally, patient characteristics of the historical ePTFE-TIPS cohort differ from the CX-TIPS cohort. Thus, the survival rates of the cohorts cannot be compared directly and the reasons for the tendentially higher 3‑year survival in the CX-TIPS cohort cannot be determined with certainty.

In conclusion, this study firstly presents the implementation of an interdisciplinary PH/TIPS board as a step towards individualized medicine in patients with decompensated cirrhosis in a large tertiary center. Secondly, it indicates the technical feasibility and clinical effectiveness of CX-TIPS in patients with PH-related complications. Thirdly, patients with risk factors such as previous overt hepatic encephalopathy, sarcopenia or cardiovascular comorbidities may be considered for underdilated CX-TIPS placement. Fourthly, PVR-TIPS represents an option for patients with PH-related complications and portal vein thrombosis.

## Supplementary Information

Figure S1. Development of decompensation events after CX-Implantation. Showing (A) the difference in the occurrence of hepatic encephalopathy and (B) the loss of ascites/bleeding control between patients with underdilated versus non-underdilated TIPS placement. *TIPS*, transjugular intrahepatic portosystemic shunt. Table S1. Comparison of patient characteristics and outcomes between patients with underdilated and non-underdilated TIPS.

## Data Availability

The data are available upon reasonable request to the corresponding author.

## Abbreviations

ACLF: Acute on chronic liver failure
ALD: Alcohol-related liver disease
BMS: Bare metal stents
COVID-19: Coronavirus disease 2019
CT: Computer tomography
CX-TIPS: Controlled-expansion transjugular intrahepatic portosystemic shunt
EASL: European Association for the Study of the Liver
EC: Ethics committee
EF-CLIF: European Foundation for the Study of Chronic Liver Failure
ePTFE: Expandable polytetrafluoroethylene
HVPG: Hepatic venous pressure gradient
IQR: Interquartile range
MELD: Model of end-stage liver disease
PH: Portal hypertension
PPG: Portal pressure gradient
ProBNP: Pro-brain-type natriuretic peptide
PVR-TIPS: Portal vein recanalization transjugular intrahepatic portosystemic shunt
SOP: Standard operating procedure
TIPS: Transjugular intrahepatic portosystemic shunt
