# Supplementary material for: Effectiveness of controlled-expansion transjugular intrahepatic portosystemic shunt (CX-TIPS) in an interdisciplinary setting at a large tertiary center
Source: Wien Klin Wochenschr. 2025 Sep 4;138(5-6):144–53. doi: 10.1007/s00508-025-02606-7 (PMC12992416; doi:10.1007/s00508-025-02606-7)
Supplement: Supplementary file 1 — Figure S1. Development of decompensation events after CX-Implantation. Showing (A) the difference in the occurrence of hepatic encephalopathy and (B) the loss of ascites/bleeding control between patients with underdilated versus non-underdilated TIPS placement. TIPS, transjugular intrahepatic portosystemic shunt. Table S1. Comparison of patient characteristics and outcomes between patients with underdilated and non-underdilated TIPS. [file 508_2025_2606_MOESM1_ESM.docx]

**Table-S1. Comparison of patient characteristics and outcomes between patients with underdilated and non-underdilated TIPS.**

*Abbreviations: ACLF, acute on chronic liver failure; AKI, acute kidney injury; ALD, alcohol-related liver disease; CTP, Child-Turcotte-Pugh-Score; BMI, body mass index; HCC, hepatocellular carcinoma; IQR, interquartile range; kg, kilogram; L, liter; m^2^, square meter; MELD, Model of End-stage Liver Disease; mL, milliliter; mmHg, millimeter of mercury; n, number; pg, picogram; PPG, portal pressure gradient; SBP, spontaneous bacterial peritonitis; TIPS, transjugular intrahepatic portosystemic shunt; µmol, micromole.*

| Patient characteristics | Underdilated TIPS (n=14) | Non-underdilated TIPS (n=89) | p-value |
| --- | --- | --- | --- |
|  |  |  |  |
| Sex, male/female (% male) | 10/4 (71.4%) | 59/30 (66.3%) | 0.704 |
| Age, years (IQR) | 58.0 (45.3-66.3) | 57.0 (49.0-66.0) | 0.574 |
| BMI, kg x m^2^ (IQR) | 26.0 (22.4-26.0) | 25.4 (21.9-29.4) | 0.744 |
|  |  |  |  |
| Etiology |  |  | 0.231 |
| ALD, n (%) | 8 (57.1%) | 55 (61.8%) |  |
| non-ALD, n (%) | 6 (42.9%) | 34 (38.2%) |  |
|  |  |  |  |
| MELD, points (IQR) | 13.2 (8.5-18.3) | 11.2 (9.9-14.9) | 0.744 |
| CTP score, points (IQR) | 8.5 (8.0-9.3) | 8.0 (7.0-8.0) | 0.426 |
| CTP stage |  |  | 0.296 |
| A, n (%) | 1 (7.1%) | 15 (16.9%) |  |
| B, n (%) | 10 (71.4%) | 65 (73.0%) |  |
| C, n (%) | 3 (21.4%) | 9 (10.1%) |  |
|  |  |  |  |
| Ammonia, µmol x L-1 (IQR) | 35.9 (25.4-55.7) | 41.4 (30.1-59.5) | 0.774 |
| proBNP, pg x mL-1 (IQR) | 221.0 (131.7-375.5) | 202.0 (85.8-567.5) | 1.000 |
|  |  |  |  |
| TIPS Indication |  |  | 0.081 |
| Bleeding, n (%) | 2 (14.3%) | 34 (38.2%) |  |
| Ascites, n (%) | 12 (85.7%) | 55 (61.8%) |  |
|  |  |  |  |
| PPG pre-TIPS, mmHg (IQR) | 17.0 (14.5-23.0) | 18.0 (15.0-22.0) | 0.730 |
| PPG post-TIPS, mmHg (IQR) | 8.0 (7.0-9.8) | 8.0 (6.0-10.0) | 0.876 |
|  |  |  |  |
| Follow-up Time, days (IQR) | 228 (57-725) | 411 (194-856) | 0.804 |
|  |  |  |  |
| Stent dilatation, n (%) | 2 (14.3%) | 11 (12.4%) | 0.840 |
| Stent reduction, n (%) | 0 (0.0%) | 5 (5.6%) | 0.363 |
|  |  |  |  |
| TIPS thrombosis, n (%) | 0 (0.0%) | 7 (7.9%) | 0.277 |
|  |  |  |  |
| Decompensation event, n (%) | 10 (71.4%) | 35 (39.3%) | **0.024** |
| Ascites complication, n (%) | 5 (35.7%) | 25 (28.1%) | 0.559 |
| Variceal bleeding, n (%) | 2 (14.3%) | 1 (1.1.%) | **0.006** |
| Hepatic encephalopathy, n (%) | 6 (42.9%) | 25 (28.1%) | 0.263 |
| ACLF, n (%) | 4 (28.6%) | 23 (25.8%) | 0.829 |
|  |  |  |  |
| Cardiac decompensation, n (%) | 2 (14.3%) | 4 (4.5%) | 0.146 |
| SBP, n (%) | 2 (14.3%) | 2 (2.2%) | **0.030** |
| AKI, n (%) | 2 (14.3%) | 11 (12.4%) | 0.840 |
|  |  |  |  |
| HCC, n (%) | 0 (0.0%) | 1 (1.1%) | 0.690 |
| Liver transplantation, n (%) | 1 (7.1%) | 10 (11.2%) | 0.645 |
| Death, n (%) | 3 (21.4%) | 22 (24.7%) | 0.790 |
| Liver-related death, n (%) | 2 (14.3%) | 16 (18.0%) | 0.735 |

**SUPPLEMENTARY FIGURE LEGENDS**

**Figure-S1. Development of decompensation events after CX-Implantation.** Showing (A) the difference in the occurrence of hepatic encephalopathy and (B) the loss of ascites/bleeding control between patients with underdilated versus non-underdilated TIPS placement.

*Abbreviations: TIPS, transjugular intrahepatic portosystemic shunt.*
